# Statin Safety in Chinese: A Population-Based Study of Older Adults

**DOI:** 10.1371/journal.pone.0150990

**Published:** 2016-03-08

**Authors:** Daniel Q. Li, Richard B. Kim, Eric McArthur, Jamie L. Fleet, Robert A. Hegele, Baiju R. Shah, Matthew A. Weir, Amber O. Molnar, Stephanie Dixon, Jack V. Tu, Sonia Anand, Amit X. Garg

**Affiliations:** 1 Division of Nephrology, Department of Medicine, Western University, London, Canada; 2 Division of Clinical Pharmacology, Department of Medicine, Western University, London, Canada; 3 Institute for Clinical Evaluative Sciences (ICES) Western, London, Ontario, Canada; 4 Blackburn Cardiovascular Genetics Laboratory, Robarts Research Institute, University of Western Ontario, London, Ontario, Canada; 5 Sunnybrook Health Sciences Centre, Toronto, Canada; 6 Division of Nephrology, Department of Medicine, University of Ottawa, Ottawa, Ontario, Canada; 7 Department of Epidemiology and Biostatistics, Western University, London, Canada; 8 Population Health Research Institute, McMaster University and Hamilton Health Sciences, Hamilton, ON, Canada; University of British Columbia, CANADA

## Abstract

**Background:**

Compared to Caucasians, Chinese achieve a higher blood concentration of statin for a given dose. It remains unknown whether this translates to increased risk of serious statin-associated adverse events amongst Chinese patients.

**Methods:**

We conducted a population-based retrospective cohort study of older adults (mean age, 74 years) newly prescribed a statin in Ontario, Canada between 2002 and 2013, where 19,033 Chinese (assessed through a validated surname algorithm) were matched (1:3) by propensity score to 57,099 non-Chinese. This study used linked healthcare databases.

**Findings:**

The follow-up observation period (mean 1.1, maximum 10.8 years) was similar between groups, as were the reasons for censoring the observation period (end of follow-up, death, or statin discontinuation). Forty-seven percent (47%) of Chinese were initiated on a higher than recommended statin dose. Compared to non-Chinese, Chinese ethnicity did not associate with any of the four serious statin-associated adverse events assessed in this study [rhabdomyolysis hazard ratio (HR) 0.61 (95% CI 0.28 to 1.34), incident diabetes HR 1.02 (95% CI 0.80 to 1.30), acute kidney injury HR 0.90 (95% CI 0.72 to 1.13), or all-cause mortality HR 0.88 (95% CI 0.74 to 1.05)]. Similar results were observed in subgroups defined by statin type and dose.

**Conclusions:**

We observed no higher risk of serious statin toxicity in Chinese than matched non-Chinese older adults with similar indicators of baseline health. Regulatory agencies should review available data, including findings from our study, to decide if a change in their statin dosing recommendations for people of Chinese ethnicity is warranted.

## Introduction

Chinese ethnicity is often associated with heightened drug sensitivity, likely due to genetic differences in drug metabolism and clearance [1, 2]. For this reason, the recommended doses of many therapeutic drugs are lower for Chinese (and others of Asian ethnicity) living in Western countries [3–5]. This recommendation extends to the use of statins, one of the most frequently prescribed drugs in the world (global users projected to reach 1 billion) [6]. Statins are associated with several rare but serious adverse events in a dose-dependent manner, including rhabdomyolysis, new-onset diabetes, and possibly acute kidney injury [7–11]. Pharmacokinetic studies have demonstrated that compared to Caucasians, Chinese achieve a higher blood concentration of statins for a given dose [12–14]. Based on this evidence, Health Canada and the US Food and Drug Administration (FDA) currently list Asian ethnicity as a risk factor for statin-induced rhabdomyolysis and recommend a lower starting and maximum dose of rosuvastatin in all Asians [15, 16]. However, it remains unclear whether Asians truly experience a higher risk of serious statin toxicity compared to non-Asians in routine practice. Previous studies have demonstrated comparable statin safety and efficacy profiles between South Asians and Caucasians living in Canada [17], while there is no clear consensus for East Asian populations living in Western countries. People of Chinese origin comprise roughly 20% of the global population and represent one of the largest minority populations in North America [18, 19]. In this population-based study in Ontario, Canada, we compared the risk of serious statin-associated adverse events in older adults of Chinese and non-Chinese origin.

## Methods

### Study Design and Setting

We conducted a population-based, retrospective cohort study at the Institute for Clinical Evaluative Sciences (ICES) according to an established protocol approved by the Research Ethics Board at Sunnybrook Health Sciences Centre (Toronto, Canada). Data on adults 66 years of age and older between June 2002 and March 2013 were obtained and analyzed through linked healthcare databases in the province of Ontario. Participant informed consent was not required for this study as all patient information was anonymized and de-identified prior to analysis. The province has about 13.6 million residents, 16% of whom are 65 years or older and have universal coverage for prescription drugs, and 4.7% of whom self-identify as Chinese [18, 20]. The reporting of this study follows guidelines for observational studies (Table A in S1 File) [21]. The date of the first prescription of a study statin served as the index date (also referred to as the cohort entry date or the date of statin initiation).

### Data Sources

We ascertained patient characteristics, drug use, covariate information and outcome data using records from five databases. The Ontario Registered Persons Database contains demographic and vital status information for all Ontario residents who have ever been issued a health card. We used the Ontario Drug Benefit database to identify prescription drug use. This database contains highly accurate records (error rate < 1%) for all outpatient prescriptions dispensed to people aged 65 years or older [22]. We identified diagnostic and procedural information on all hospitalizations from the Canadian Institute for Health Information Discharge Abstract Database (CIHI-DAD). We obtained covariate information from the Ontario Health Insurance Plan (OHIP) database, which includes fee-for-service health claims for inpatient and outpatient physician services. Finally, we identified new onset diabetes from the Ontario Diabetes Database (ODD) [23]. We have previously used these databases to research adverse drug events (including outcomes of statin toxicity and health services) [24–30]. We used *International Classification of Diseases*, *9th revision* (*ICD-9*; pre-2002) and *10th revision* (*ICD-10*; post-2002) codes to assess baseline comorbidities in the three years prior to the index date (Table B in S1 File). The codes we used to ascertain outcomes are detailed in Table C in S1 File, which lists only *ICD-10* codes as this was the only coding system in use during the follow-up period.

### Classification by Chinese Ethnicity

We identified patients of Chinese and non-Chinese ethnicity using a list of 1,133 unique Chinese surnames prior to anonymization of linked healthcare data. This surname list was developed and validated in Ontario, and has a sensitivity of 80% and a positive predictive value of 92% in classifying individuals who self-identify as Chinese [31]. While people of other Asian ethnicities are included in the non-Chinese group, the proportion is likely small as 77% of the Ontario population does not belong to a visible ethnic minority [32].

### Patients

We established a cohort of older adults (i.e. > 65 years) in Ontario, Canada, who initiated one of three study statins (atorvastatin, rosuvastatin, or simvastatin). Other drugs in this class are infrequently used in Ontario, comprising less than 6% of all statin prescriptions during the study period. We excluded patients if they met any of the following criteria: (a) they were in their first year of eligibility for prescription drug coverage (aged 65 years), to avoid incomplete medication records; (b) they received a prescription for any statin (study or non-study) in the 180 days prior to the index date, to confirm new statin use; (c) they were without healthcare eligibility in the 3 years prior to index date (for reasons such as immigration), to ensure sufficient data to assess baseline comorbidities, and (d) they had co-prescriptions for potent CYP3A4 inhibitors (protease inhibitors, chloramphenicol, and antifungals) in the 180 days prior to their index date, to avoid possible statin toxicity from drug-drug interactions [33]. A patient could enter the cohort only once. To select statin users with similar characteristics, Chinese were matched 1:3 to non-Chinese on the following baseline characteristics: age (within two years), sex, index date (same year), chronic kidney disease, coronary artery disease, statin type and dose (high dose defined as ≥10 mg rosuvastatin, ≥20 mg atorvastatin, or ≥40 mg simvastatin; other values were classified as low dose [8]), and the logit of the propensity score for the predicted probability of Chinese ethnicity (within 0.2 standard deviations). The propensity score was derived from a logistic regression model containing 58 appropriately selected baseline variables [34] (Table 1). To measure baseline comorbidity, we used two scoring systems: the Adjusted Clinical Group (ACG) score and the Charlson Comorbidity Index [35–37].

**Table 1:** Characteristics of Chinese and non-Chinese at cohort entry.

		Unmatched				Matched		
		Chinese	Non-Chinese			Chinese	Non-Chinese	
		N = 20,598 (%)	N = 605,175 (%)		Standardized Difference *	N = 19,033 (%)	N = 57,099 (%)	Standardized Difference *
**Demographics**								
Age, mean ± SD		73.6 ± 6.3	73.8 ± 6.5	3%		73.3 ± 6.0	73.3 ± 6.0	0%
Women		11,094 (54)	318,527 (53)	2%		10,260 (54)	30,780 (54)	0%
Income Quintile †								
	One (lowest)	4,489 (22)	118,328 (20)	6%		4,025 (21)	12,075 (21)	0%
	Two	4,919 (24)	127,257 (21)	7%		4,490 (24)	13,796 (24)	1%
	Three (middle)	3,923 (19)	119,207 (20)	2%		3,711 (19)	11,072 (19)	0%
	Four	3,854 (19)	118,085 (20)	2%		3,622 (19)	10,752 (19)	1%
	Five (highest)	3,362 (16)	120,476 (20)	9%		3,185 (17)	9,404 (16)	1%
Rural Residence		162 (1)	87,397 (14)	53%		154 (1)	630 (1)	3%
Long-term Care Facility		483 (2)	13970 (2)	0.2%		402 (2)	827 (1)	5%
Year of Cohort Entry ‡								
	2002–2003	3,183 (15)	110,941 (18)	8%		3052 (16)	9,156 (16)	0%
	2004–2005	3,663 (18)	120,927 (20)	6%		3,443 (18)	10,329 (18)	0%
	2006–2007	4,007 (19)	110,266 (18)	3%		3,616 (19)	10,848 (19)	0%
	2008–2009	3,838 (19)	106,714 (18)	3%		3,473 (18)	10,410 (18)	0%
	2010–2011	3,962 (19)	102,268 (17)	6%		3,656 (19)	10,968 (19)	0%
	2012	1,945 (9)	54,090 (9)	2%		1,796 (9)	5,388 (9)	0%
**Health care use** §								
Hospital discharge in the two days prior		897 (4)	60,918 (10)	22%		822 (4)	2,332 (4)	1%
Cardiologist visits								
	0	13,348 (65)	334,923 (55)	19%		12,443 (65)	36,689 (64)	2%
	1	1,210 (6)	32,991 (5)	2%		1,120 (6)	3,573 (6)	2%
	2	1,065 (5)	39,043 (6)	5%		9,72 (5)	3,287 (6)	3%
	≥3	4,975 (24)	198,218 (33)	19%		4,498 (24)	13,550 (24)	0%
Family physician visits								
	0	582 (3)	21,030 (3)	4%		565 (3)	1,933 (3)	2%
	1	436 (2)	18,127 (3)	6%		423 (2)	1,638 (3)	4%
	2	609 (3)	23,206 (4)	5%		577 (3)	2,279 (4)	5%
	≥3	18,971 (92)	542,812 (90)	8%		17,468 (92)	51,249 (90)	7%
Hospitalizations								
	0	16,813 (82)	414,679 (69)	31%		15,551 (82)	45,896 (80)	3%
	1	2,569 (12)	116,362 (19)	19%		2,364 (12)	8,053 (14)	5%
	2	841 (4)	45,439 (8)	15%		7,82 (4)	2,287 (4)	1%
	≥3	375 (2)	28,695 (5)	16%		3,36 (2)	863 (2)	2%
ER visits								
	0	17,044 (83)	382,661 (63)	45%		15,750 (83)	46,171 (81)	5%
	1	2,523 (12)	132,761 (22)	26%		2,340 (12)	8,202 (14)	6%
	2	678 (3)	47,828 (8)	20%		635 (3)	1,930 (3)	0%
	≥3	353 (2)	41,925 (7)	26%		308 (2)	796 (1)	2%
Coronary artery bypass grafting (CABG)		283 (1)	23,632 (4)	16%		275 (1)	769 (1)	1%
Holter monitoring		1,067 (5)	43,793 (7)	9%		993 (5)	3,022 (5)	0%
Cardiac Stress Test		1,941 (9)	85,821 (14)	15%		1,823 (10)	5,191 (9)	2%
Echocardiography		3,503 (17)	13,4553 (22)	13%		3,160 (17)	9,211 (16)	1%
Pulmonary function test		1,099 (5)	50,967 (8)	12%		1,049 (6)	3,087 (5)	1%
Cholesterol test		17,106 (83)	444,422 (73)	23%		15,722 (83)	47,540 (83)	2%
**Co-morbidities** ||								
Charlson comorbidity index #								
	0	17,927 (87)	468,821 (77)	25%		16,652 (87)	49,963 (88)	0%
	1	1,176 (6)	60,024 (10)	16%		1,108 (6)	3,314 (6)	0%
	2	742 (4)	38,237 (6)	13%		671 (4)	1,976 (3)	0%
	≥3	753 (4)	38,093 (6)	12%		602 (3)	1,846 (3)	0%
Johns Hopkins ACG score								
	0–4	10,312 (50)	260,430 (43)	14%		9,718 (51)	29,795 (52)	2%
	5–9	8,697 (42)	272,385 (45)	6%		7,912 (42)	23,640 (41)	0%
	10–14	1,497 (7)	65,918 (11)	13%		1,329 (7)	3,472 (6)	4%
	≥15	92 (0.4)	6,442 (1)	7%		74 (0.4)	192 (0.3)	1%
Chronic kidney disease		1,232 (6)	34,428 (6)	1%		732 (4)	2,196 (4)	0%
Major cancer **		1,297 (6)	61,331 (10)	14%		1,207 (6)	3,772 (7)	1%
Coronary artery disease††		3,309 (16)	175,078 (29)	31%		2,885 (15)	8,655 (15)	0%
Peripheral vascular disease		70 (0.3)	9,010 (1)	12%		53 (0.3)	380 (1)	6%
Diabetes‡‡		5,356 (26)	134,439 (22)	9%		4,813 (25)	14,583 (26)	1%
Chronic liver disease		1,263 (6)	12,219 (2)	21%		762 (4)	2,209 (4)	1%
Stroke/TIA		601 (3)	26,045 (4)	7%		559 (3)	1,329 (2)	4%
Heart failure		963 (5)	61,069 (10)	21%		834 (4)	2,544 (4)	0%
Sepsis		260 (1)	10,025 (2)	3%		220 (1)	576 (1)	1%
Hypertension		11,360 (55)	383,613 (63)	17%		10,291 (54)	31,381 (55)	2%
Angina		1,669 (8)	114,145 (19)	32%		1,587 (8)	4,356 (8)	3%
Arrhythmia		580 (3)	39,899 (7)	18%		508 (3)	1,564 (3)	0%
**Medications** §§								
ACE Inhibitors		5,066 (25)	253,669 (42)	37%		4,838 (25)	14,004 (25)	2%
ARBs		5,445 (26)	95,466 (16)	26%		4,572 (24)	14,187 (25)	2%
Beta blockers		5,120 (25)	198,080 (33)	17%		4,616 (24)	13,421 (24)	2%
Calcium channel blockers		6,969 (34)	170,265 (28)	12%		6,047 (32)	18,584 (33)	2%
Loop diuretics		873 (4)	60,254 (10)	22%		718 (4)	2,378 (4)	2%
Potassium sparing diuretics		506 (2)	33,360 (6)	16%		477 (3)	1,475 (3)	0%
NSAIDs		3,216 (16)	108,378 (18)	6%		2,964 (16)	10,176 (18)	6%
Thiazide diuretics		2,576 (13)	121,829 (20)	21%		2,438 (13)	7,558 (13)	1%
Anticholinergics		543 (3)	27,886 (5)	11%		483 (3)	1,577 (3)	1%
Corticosteroids		892 (4)	29,894 (5)	3%		819 (4)	2,364 (4)	1%
Beta2-agonists		1,307 (6)	62,033 (10)	14%		1,203 (6)	3,757 (7)	1%
Amiodarone		150 (1)	9,320 (2)	8%		133 (1)	343 (1)	1%
Cyclosporine		13 (0.1)	209 (0.0)	1%		9 (0.0)	22 (0.0)	0%
Warfarin		796 (4)	47,906 (8)	17%		721 (4)	2,114 (4)	0%
Antidepressants		901 (4)	65,249 (11)	24%		846 (4)	2,570 (5)	0%
Levothyroxine		1,155 (6)	73,777 (12)	23%		1,098 (6)	3,330 (6)	0%
Quetiapine		67 (0.3)	3,803 (0.6)	4%		63 (0.3)	231 (0.4)	1%
Riseperidone		80 (0.4)	3,962 (0.7)	4%		69 (0.4)	239 (0.4)	1%
Olanzapine		67 (0.3)	3,252 (0.6)	3%		62 (0.3)	217 (0.4)	1%
**Statin daily dose, median [IQR], mg**								
Atorvastatin		10 [10–20]	10 [10–20]			10 [10–20]	10 [10–20]	
Rosuvastatin		5 [5–10]	10 [10–10]			5 [5–10]	5 [5–10]	
Simvastatin		20 [10–20]	20 [10–40]			20 [10–20]	20 [10–20]	
**Rosuvastatin starting dose** ||||								
0 - <5 mg/day		195 (0.9)	2,018 (0.3)			158 (0.9)	298 (0.5)	
5 - <10 mg/day		3,594 (17)	43,137 (7)			3,075 (16.2)	8,350 (14.6)	
10 - <15 mg/day		11,128 (54)	306,722 (51)			10,491 (55.1)	31,003 (54.3)	
15 - <20 mg/day		50 (0.2)	1,103 (0.2)			47 (0.3)	96 (0.2)	
≥20 mg/day		5,631 (27)	252,195 (42)			5262 (27.7)	17,352 (30.4)	

***Abbreviations***: TIA, transient ischemic attack; ACE, angiotensin-converting enzyme; ACG, adjusted clinical groups; ARB, angiotensin II receptor blocker; NSAID, nonsteroidal anti-inflammatory drug; SD, standard deviation; IQR, interquartile range.

* Standardized differences are less sensitive to sample size than traditional hypothesis tests. They provide a measure of the difference between groups divided by the pooled standard deviation; a value greater than 10% is interpreted as a meaningful difference between the groups.

† Income was categorized into fifths of average neighborhood income on the index date.

‡ The date of cohort entry is also referred to as the index date.

§ Assessed by administrative database codes in the previous 1 year (unless otherwise specified).

|| Assessed by administrative database codes in the previous 3 years.

# Charlson comorbidity index [34, 35] was calculated using 3 years of hospitalization data. No prior hospitalizations received a score of 0.

** Major cancers include esophagus, lung, bowel, liver, pancreas, breast, male/female reproductive organs, as well as leukemia and lymphomas.

†† Coronary artery disease includes both diagnoses of angina and coronary artery revascularization.

‡‡ Assessed using prescriptions for diabetic medications.

§§ Assessed in the previous 120 days.

|||| Health Canada recommends a starting rosuvastatin dose of 5mg for individuals of Asian ethnicity.

### Outcomes

We examined four outcomes associated with statin toxicity as specified in previous studies [7, 10, 24–27, 29, 30]: hospitalization with rhabdomyolysis, incident diabetes, hospitalization with acute kidney injury, and all-cause mortality (the diagnostic codes and their validity are presented in Table C in S1 File). For incident diabetes outcome, we excluded matched sets of patients with evidence of baseline diabetes. We selected acute kidney injury as a potential adverse event of statin toxicity based on a recent report [8]; while completing our study, additional information became available questioning whether acute kidney injury is truly a potential adverse event of a higher serum statin concentration [38, 39]. While liver injury is a historic concern linked to statin therapy, recent studies have not confirmed this association and regulatory bodies no longer identify it as a major concern [40, 41]; thus we decided *a priori* not to include it as a study outcome. Since up to 25 unique diagnostic codes can be assigned per hospitalization, patients with multiple diagnosis codes were accounted for in the assessment of each hospitalization outcome. However, the overall incidence is underestimated due to a spectrum bias in hospital diagnosis coding, particularly for milder forms of conditions. For example, the incidence of acute kidney injury can be underestimated up to five-fold when assessed by diagnostic codes compared to laboratory values [27].

### Statistical Analyses

We compared baseline characteristics of Chinese and non-Chinese using standardized differences [42]. This metric describes differences between group means relative to the pooled standard deviation and is considered a clinically meaningful difference if greater than 10%. We used Cox proportional hazard regression analyses to estimate hazard ratios and 95% confidence intervals for each of our three outcomes (stratifying the models on matched sets). We considered a two-tailed p-value of less than 0.05 to be statistically significant. In the follow-up for these three outcomes, we censored the observation period at the time of death, statin discontinuation, or end of database records (March 31, 2013). In the primary analysis, statin discontinuation was defined by no evidence of a repeat prescription for the same statin within 30 days following the end of the previous prescription day supply. In our data sources, any deaths, dispensed medications, and associated dates are recorded with high accuracy [22, 43]. We repeated the primary analysis in subgroups defined by statin type (atorvastatin, rosuvastatin, or simvastatin) and statin dose (high *vs*. low, as previously defined). We conducted all statistical analyses using SAS version 9.3 (SAS Institute, Cary, North Carolina).

## Results

### Baseline Characteristics and Follow-up Observation Period

Cohort selection is presented in Fig A in S1 File. We identified 625,363 new statin users aged 66 or older who were Chinese (n = 20,598) or non-Chinese (n = 604,765). After 1:3 matching we retained 76,132 individuals (19,033 Chinese, 57,099 non-Chinese). The baseline characteristics of patients before and after matching are presented in Table 1. Before matching, those who were Chinese exhibited significant differences in several baseline characterstics and were generally healthier compared to non-Chinese. While Chinese users were more likely to be prescribed lower doses of rosuvastatin than non-Chinese (Table 1), more than half of Chinese were given a starting dose higher than 5 mg per day for each year of the study period, which is contrary to the recommendation from Health Canada and US Food and Drug Administration on rosuvastatin dosing in Asians released in 2005 (Fig B in S1 File). Matching resulted in two groups similar in age, sex, year of statin prescription, statin type, statin dose, baseline healthcare usage, and multiple other baseline characteristics (Table 1).

The mean length of follow-up was 1.1 years and was similar between the groups (1.3 years in Chinese, 1.1 years in non-Chinese; maximum 10.8 years). The total person-years of follow-up was 85,893 (24,259 Chinese, 61,634 non-Chinese). Statin discontinuation during follow-up was lower for Chinese compared to non-Chinese (Table D in S1 File).

### Outcomes

**Fig 1:**
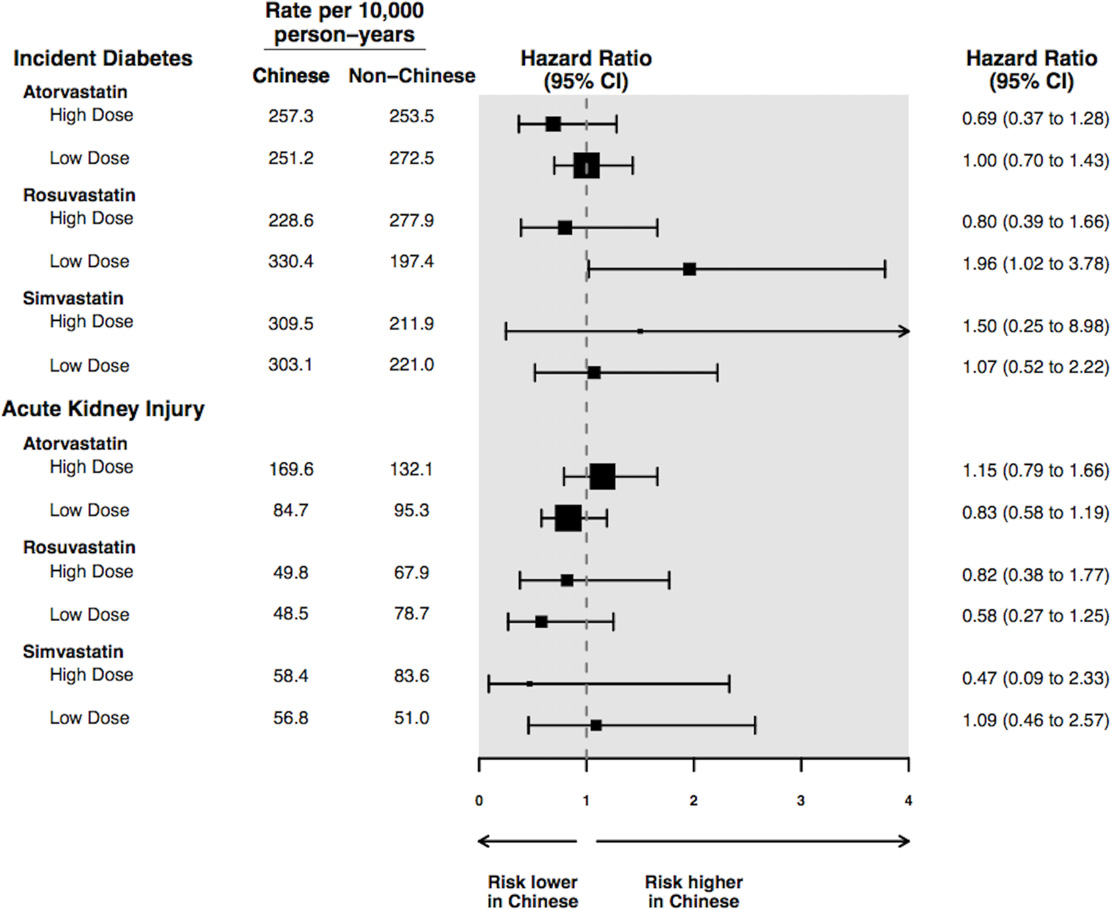
Subgroup analysis by statin type and dose for hospitalization with acute kidney. injury and a diagnosis of diabetes mellitus *†. * High dose defined as ≥10 mg rosuvastatin, ≥20 mg atorvastatin, or ≥40 mg simvastatin; other values classified as low dose. † To comply with privacy regulations numerators of 1 to 5 cannot be reported individually; for this reason we simply present the rate per 10,000 person-years, without additional information on the number of events in each subgroup.

Study outcomes are shown in Table 2. Results are expressed in events per 10,000 person-years, as well as in hazard ratios with patients of non-Chinese ethnicity serving as the referent group. There was no significant difference between Chinese *vs*. non-Chinese in the risk of hospitalization with rhabdomyolysis (hazard ratio [HR] 0.61, 95% confidence interval [CI] 0.28 to 1.34, p = 0.22), incident diabetes (HR 1.02, 95% CI 0.80 to 1.30, p = 0.89), hospitalization with acute kidney injury (HR 0.90, 95% CI 0.72 to 1.13, p = 0.36), or all-cause mortality (HR 0.88, 95% CI 0.74 to 1.05, p = 0.17). We tested the proportionality assumption for all four outcomes using time-dependent covariates in the Cox proportional hazards regression model. The proportionality assumption was not violated for any of the outcomes, with all p-values greater than 0.05. Additionally, we treated death as a competing risk and the results did not differ (Table E in S1 File). The results of subgroup analyses are shown in Fig 1. The risk of rhabdomyolysis was not assessed in subgroups because there were too few events for meaningful analysis. Statin type and dose did not significantly modify the association between Chinese ethnicity and other outcomes. While there was a higher risk of incident diabetes in Chinese patients taking a lower dose of rosuvastatin, the interaction of incident diabetes with higher dose rosuvatatin was not statistically significant (p = 0.07).

**Table 2:** Statin-associated adverse outcomes in Chinese and non-Chinese (referent).

	Number of Events (rate per 10,000 person-years)		Hazard Ratio (95% CI)	P-value
	Chinese (n = 19,033)	Non-Chinese (n = 57,099)		
**Rhabdomyolysis** *	16 (6.6)	79 (12.8)	0.61 (0.28 to 1.34)	0.22
**Incident diabetes** †	182 (267.5)	475 (252.4)	1.02 (0.80 to 1.30)	0.89
**Acute kidney injury** *	215 (89.2)	556 (90.8)	0.90 (0.72 to 1.13)	0.36
**All-cause mortality** ‡	324 (133.5)	965 (156.4)	0.88 (0.74 to 1.05)	0.17

***Abbreviations***: confidence interval (CI)

* Outcomes assessed with hospital diagnosis codes. This underestimates the true event rate because these codes have high specificity but low sensitivity.

† Diabetes diagnoses assessed with the Ontario Diabetes Database; only matched sets where none of the users had evidence of baseline diabetes were included in this analysis (6,170 Chinese, 18,510 non-Chinese).

‡ All-cause mortality assessed with vital status field of Registered Persons Database of Ontario.

### Additional analyses

We conducted several additional analyses after knowledge of the primary results. First, if prescribing physicians titrated the dose of statin differently among Chinese and non-Chinese (to clinical effects such as lipid level), this could abrogate the effect of ethnicity. To assess this we examined the change in statin dose over the course of follow-up and expressed the change standardized to the initial dose ([final dose–initial dose in follow-up] / initial dose × 100). We observed very little change in dose over the follow-up in both Chinese and non-Chinese groups: the median (interquartile range) change in dose for both groups was 0 (0 to 0). We also recorded the frequency and direction of dose changes within the cohort. Doses were stable in 84.0% in Chinese and 86.8% in non-Chinese, and increased in 11.5% of Chinese and 10.4% of non-Chinese.

Second, although we observed no higher rate of statin discontinuation in Chinese versus non-Chinese, it is possible that outpatient blood monitoring differed in the two groups and influenced outcomes. Outpatient blood testing (which is a billable service to the universal provincial healthcare plan) is accurately recorded in our data sources (but values are not available). We observed no significant difference between the two groups in the proportion of those with creatine kinase, serum creatinine, serum glucose, or glycated hemoglobin testing during the follow-up periods (Table F in S1 File).

Third, in the primary analysis statin discontinuation was defined as absence of a prescription renewal within 30 days. In an additional analysis, we lengthened this definition to 100 days (which is the maximum day supply dispensed for a given prescription in Ontario). While the mean follow-up increased to 2.5 years, there remained no higher risk of any of our study outcomes in Chinese compared to non-Chinese (Table G in S1 File).

## Discussion

Based on evidence of higher blood levels of a given statin in Asians, regulatory agencies such as Health Canada and the US Food and Drug Administration recommend a lower dose of statins for Asians compared to non-Asians. Although we showed that this recommendation is not frequently followed for Chinese patients in routine care, we observed no higher risk of statin toxicity in Chinese than matched non-Chinese older adults with similar indicators of baseline health.

What is the basis for the recommended statin reduction in Chinese? Historically this was attributed to the smaller body size and weight of Asians, as well as environmental and dietary differences [3, 15], but pharmacokinetic modeling and clinical studies do not support these as causative factors [44]. In recent years, a growing number of pharmacogenomic studies describe significant differences between Chinese and non-Chinese in the frequencies of common genetic polymorphisms linked to statin metabolism and transport [12]. For example, the frequency of the single nucleotide polymorphism (SNP) c.421 C>A in the *ABCG2* gene (encoding an efflux drug transporter) is much higher in the Chinese population (35% in Chinese versus 15% in Caucasians), which is associated with increased systemic exposure to rosuvastatin [44]. Interestingly, the presence of these genetic polymorphisms have also been linked to enhanced lipid lowering effect, likely due to higher hepatic concentration of substrate statins [45].

A higher blood concentration with a given dose of statin in Chinese versus non-Chinese is clear [13–15]. However, the risk of severe statin-associated adverse events associated with Chinese ethnicity remains unclear. In many large randomized controlled trials conducted in both Asian and Western countries, amongst Asians there is no reported difference in the safety or tolerability of a lower compared to higher dose of statin [3, 41, 46–48]. Rather, above-standard doses of statins provided additional cholesterol-lowering benefit in Asians [14]. Regulatory agencies should review available data, including findings from our study, to see if a change in their statin dosing recommendations for people of Chinese ethnicity (and Asian ethnicity in general) is warranted.

Our study’s findings must be interpreted in the context of its limitations. First, we identified patients as Chinese or non-Chinese based on a list of uniquely Chinese surnames, rather than self-reported ethnicity or genetic assessment. While the surname algorithm has been validated and used in other health outcome studies, misclassification is possible. Second, some outcomes were assessed with hospital diagnosis codes, and milder forms of adverse events may have been missed. In HPS2-THRIVE, a large placebo-controlled trial of 25,673 high-risk patients, Chinese patients taking simvastatin 40 mg had a higher absolute risk of myopathy than patients in Europe [49]. The outcome in HPS2-THRIVE included milder symptoms of myopathy, which differs from the focus in this study (i.e. serious statin-associated adverse events that resulted in hospitalization). Third, although our study outcomes were chosen based on evidence from previous literature, we appreciate that some outcomes remain controversial in their association with statin use. Fourth, it is important to note that risk profiling based on ethnicity may not generalize well to the care of an individual. Even within a population or ethnic group, there exist a mixture of genotypes that serve as determinants of drug exposure and response [1]. Other inter-individual factors that are not well characterized may also affect overall drug responses; for example in this study we did not have information on body composition or baseline laboratory values, nor did we account for dietary differences and the use of non-prescription drugs. Some common ingredients found in traditional Chinese medicine and the Chinese diet, such as red yeast rice, may adversely interact with statins [50, 51]. Fifth, it is difficult to objectively measure and compare drug compliance in retrospective observational studies. While we were able to compare the rates of statin discontinuation (which was lower in Chinese vs. non-Chinese), the exact reasons for discontinuation were unclear. Finally, we only studied older adults given our available data. However, those who are younger (and often healthier) would be expected to have lower rates of statin toxicity, making it more difficult to observe a meaningful difference between Chinese and non-Chinese patients [52].

## Conclusion

We observed no higher risk of serious statin toxicity in Chinese than matched non-Chinese older adults with similar indicators of baseline health. Regulatory agencies should review available data to decide if a change in their statin dosing recommendations for people of Chinese ethnicity is warranted.

## Supporting Information

S1 File Supporting Information. Table A. STROBE checklist. Table B. Coding definitions for demographic and comorbid conditions. Table C. Coding definitions for hospitalization with rhabdomyolysis, incident diabetes, and hospitalization with acute kidney injury. Table D. Reasons the observation time was censored amongst the matched cohort. Table E. Statin-associated adverse outcomes in Chinese and non-Chinese (referent) adjusting for competing risk of death. Table F: Outpatient blood testing during follow-up. Table G. Statin-associated adverse outcomes in Chinese and non-Chinese (referent) with at least 100 days grace period for subsequent statin prescription. Fig A. Flow diagram of cohort selection. Fig B. Total number of Chinese newly dispensed rosuvastatin each year and proportion with ≥10mg/day starting dose.
(DOC)
