# Supplementary material for: Statin Safety in Chinese: A Population-Based Study of Older Adults
Source: PLoS One. 2016 Mar 8;11(3):e0150990. doi: 10.1371/journal.pone.0150990 (PMC4783028; doi:10.1371/journal.pone.0150990)
Supplement: S1 File — Table A. STROBE checklist. Table B. Coding definitions for demographic and comorbid conditions. Table C. Coding definitions for hospitalization with rhabdomyolysis, incident diabetes, and hospitalization with acute kidney injury. Table D. Reasons the observation time was censored amongst the matched cohort. Table E. Statin-associated adverse outcomes in Chinese and non-Chinese (referent) adjusting for competing risk of death. Table F: Outpatient blood testing during follow-up. Table G. Statin-associated adverse outcomes in Chinese and non-Chinese (referent) with at least 100 days grace period for subsequent statin prescription. Fig A. Flow diagram of cohort selection. Fig B. Total number of Chinese newly dispensed rosuvastatin each year and proportion with ≥10mg/day starting dose. (DOC) [file pone.0150990.s001.doc]

**Supporting Information**

**Table A. STROBE checklist.**

| **STROBE checklist** | | | | |
| --- | --- | --- | --- | --- |
|  | **Item No** | | **Recommendation** | **Reported** |
| **Title and abstract** | 1 | | (a) Indicate the study’s design with a commonly used term in the title or the abstract | Abstract |
|  |  |  | (b) Provide in the abstract an informative and balanced summary of what was done and what was found | Abstract |
| **Introduction** | | | |  |
| Background / rationale | 2 | | Explain the scientific background and rationale for the investigation being reported | Introduction |
| Objectives | 3 | | State specific objectives, including any pre-specified hypotheses | Introduction |
| **Methods** | | | |  |
| Study design | 4 | | Present key elements of study design early in the paper | Methods |
| Setting | 5 | | Describe the setting, locations, and relevant dates, including periods of recruitment, exposure, follow-up, and data collection | Methods |
| Participants | 6 | | (a) Give the eligibility criteria, and the sources and methods of selection of participants. Describe methods of follow-up | Methods |
|  |  |  | (b) For matched studies, give matching criteria and number of exposed and unexposed | Methods |
| Variables | 7 | | Clearly define all outcomes, exposures, predictors, potential confounders, and effect modifiers. Give diagnostic criteria, if applicable | Methods  eTable 2  eTable 3 |
| Data sources/ measurement | 8 | | For each variable of interest, give sources of data and details of methods of assessment (measurement). Describe comparability of assessment methods if there is more than one group | Methods  eTable 2  eTable 3 |
| Bias | 9 | | Describe any efforts to address potential sources of bias | Methods |
| Study size | 10 | | Explain how the study size was arrived at | Methods |
| Quantitative variables | 11 | | Explain how quantitative variables were handled in the analyses. If applicable, describe which groupings were chosen and why | Methods |
| Statistical methods | 12 | | (a) Describe all statistical methods, including those used to control for confounding | Methods |
|  |  |  | (b) Describe any methods used to examine subgroups and interactions | Methods |
|  |  |  | (c) Explain how missing data were addressed | Not applicable (N/A) |
|  |  |  | (d) If applicable, explain how loss to follow-up was addressed | Methods |
|  |  |  | (e) Describe any sensitivity analyses | N/A |
| **Results** | | | |  |
| Participants | 13 | (a) Report numbers of individuals at each stage of study—e.g. numbers potentially eligible, examined for eligibility, confirmed eligible, included in the study, completing follow-up, and analyzed | | Results  eFigure 1 |
|  |  | (b) Give reasons for non-participation at each stage | | N/A |
|  |  | (c) Consider use of a flow diagram | | eFigure 1 |
| Descriptive data | 14 | (a) Give characteristics of study participants (e.g. demographic, clinical, social) and information on exposures and potential confounders | | Results  Table 1  eTable 2  eTable 4 |
|  |  | (b) Indicate number of participants with missing data for each variable of interest | | N/A |
|  |  | (c) Summarize follow-up time (e.g. average and total amount) | | Results |
| Outcome data | 15 | Report numbers of outcome events or summary measures over time | | Results  Table 2 |
| Main results | 16 | (a) Give unadjusted estimates and, if applicable, confounder-adjusted estimates and their precision (e.g. 95% confidence interval). Make clear which confounders were adjusted for and why they were included | | Results  Table 2 |
|  |  | (b) Report category boundaries when continuous variables were categorized | | Table 1 |
|  |  | (c) If relevant, consider translating estimates of relative risk into absolute risk for a meaningful time period | | N/A |
| Other analyses | 17 | Report other analyses done—e.g. analyses of subgroups and interactions, and sensitivity analyses | | Results  Figure 1 |
| **Discussion** | | | |  |
| Key results | 18 | Summarize key results with reference to study objectives | | Discussion |
| Limitations | 19 | Discuss limitations of the study, taking into account sources of potential bias or imprecision. Discuss both direction and magnitude of any potential bias | | Discussion |
| Interpretation | 20 | Give a cautious overall interpretation of results considering objectives, limitations, multiplicity of analyses, results from similar studies, and other relevant evidence | | Discussion |
| Generaliza-bility | 21 | Discuss the generalizability (external validity) of the study results | | Discussion |
| **Other information** | | | |  |
| Funding | 22 | Give the source of funding and the role of the funders for the present study and, if applicable, for the original study on which the present article is based | | Disclosure |

**Table B. Coding definitions for demographic and comorbid conditions.**

| **Characteristics** | **Database** | **Codes** |
| --- | --- | --- |
| Age | RPDB |  |
| Sex | RPDB |  |
| Socioeconomic status | Statistics Canada |  |
| Chronic Kidney Disease | CIHI-DAD  OHIP | **ICD-9** 4030, 4031, 4039, 4040, 4041, 4049, 585, 586. 5888, 5889, 25040.  **ICD-10** E102, E112, E132, E142, I12, I13, N08, N18, N19.  403, 585. |
| Major Cancers | CIHI-DAD  OHIP | **ICD-9** 150, 154, 155, 157, 162, 174, 175, 185, 203, 204, 205, 206, 207, 208.  **ICD-10** 971, 980, 982, 984, 985, 986, 987, 988, 989, 990, 991, 993, C15, C18, C19, C20, C22, C25, C34, C50, C56, C61, C82, C83, C85, C91, C92, C93, C94, C95, D00, D05.  203, 204, 205, 206, 207, 208, 150, 154, 155, 157, 162, 174, 175, 183, 185. |
| Coronary Artery Disease | CIHI-DAD  OHIP | **ICD-9** 412, 410, 414, 4292, 4295, 4296, 4297.  **ICD-10** I21, I22, I23, I24, I25, Z955, Z958, Z959, R931, T822.  **CCI** 1IJ26, 1IJ27, 1IJ54, 1IJ57, 1IJ50, 1IJ76.  **CCP** 4801, 4802, 4803, 4804, 4805, 481, 482, 483.  R741, R742, R743, G298, E646, E651, E652, E654, E655, G262, Z434, Z448, 410, 412. |
| Peripheral Vascular Disease | CIHI-DAD  OHIP | **ICD-9** 4402, 4408, 4409, 5571, 4439, 444.  **ICD-10** I700, I702, I708, I709, I731, I738, I739, K551.  **CCP** 5125, 5129, 5014, 5016, 5018, 5028, 5038.  **CCI** 1KA76, 1KA50, 1KE76, 1KG26, 1KG50, 1KG57, 1KG76MI, 1KG87.  R787, R780, R797, R804, R809, R875, R815, R936, R783, R784, R785, E626, R814, R786, R937, R860, R861, R855, R856, R933, R934, R791, E672, R794, R813, R867, E649. |
| Chronic Liver Disease | CIHI-DAD | **ICD-9** 4561, 4562, 070, 5722, 5723, 5724, 5728, 573, 7824, V026, 2750, 2751, 7891, 7895, 571.  **ICD-10** B16, B17, B18, B19, I85, R17, R18, R160, R162, B942, Z225, E831, E830, K70, K713, K714, K715, K717, K721, K729, K73, K74, K753, K754, K758, K759, K76, K77. |
| Stroke/Transient Ischemic Attack | CIHI-DAD | **ICD-9** 430, 431, 434, 435, 436.  **ICD-10** I630, I631, I632, I633, I634, I635, I638, I639, I64, H341, I600, I601, I602, I603, I604, I605, I606, I607, I609, I61, G450, G451, G452, G453, G458, G459. |
| Heart Failure | CIHI-DAD  OHIP | **ICD-9** 425, 5184, 514, 428.  **ICD-10** I500, I501, I509, I255, J81. CCP 4961, 4962, 4963, 4964.  **CCI** 1HP53, 1HP55, 1HZ53GRFR, 1HZ53LAFR, 1HZ53SYFR.  R701, R702, Z429, 428. |
| Sepsis | CIHI-DAD  OHIP | **ICD-9** 0031, 0380, 0381, 0382, 0384, 0388, 0389, 0545.  **ICD-10** A40, A41, R572.  038. |
| Hypertension | CIHI-DAD | **ICD9** 401, 402, 403, 404, 405.  **ICD10** I10, I11, I12, I13, I15. |
| Angina | CIHI-DAD  OHIP | **ICD-9** 413.  **ICD-10** I20, I23.  413. |
| Aortic aneurysm repair/bypass | CCI  CCP  OHIP | 1KA76.  5024, 5034.  R802, R816, R817, R783, R784, R785, R814. |
| Arrhythmia | CIHI-DAD  OHIP | **ICD-9** 4261, 4262, 4263, 4264, 4265, 4266, 4267, 4268, 4269, 427, 7850.  **ICD-10** I48, I44, I45, I47, I4900, I4901, I491, I492, I493, I494, I498, I499, R000, R001.  G178, G179, G249, G261, G259, Z443, Z431, Z437. |

***Abbreviations***: CCI = Canadian Classification of Health Interventions; CCP = Canadian Classification of Diagnostic, Therapeutic and Surgical Procedures; CIHI DAD = Canadian Institute for Health Information Discharge Abstract Database; ICD-9 = International Classification of Diseases, Ninth Revision; ICD-10 = International Classification of Diseases, Tenth Revision; OHIP = Ontario Health Insurance Plan; RPDB = Registered Persons Database of Ontario

**Table C. Coding definitions for hospitalization with rhabdomyolysis, incident diabetes, and hospitalization with acute kidney injury.**

| **Condition** | **Database** | **Code** |
| --- | --- | --- |
| Rhabdomyolysis * | CIHI-DAD | ICD-10 M628, T296, G210 |
| Incident Diabetes † | ODD |  |
| Acute Kidney Injury ‡ | CIHI-DAD | ICD-10 N17 |
| All-cause mortality § | RPDB | Vital status field |

***Abbreviations***: CIHI DAD = Canadian Institute for Health Information Discharge Abstract Database; ICD-10 = International Classification of Diseases, Tenth Revision; ODD = Ontario Diabetes Database; RPDB = Registered Persons Database of Ontario

***** In Ontario, we have previously observed that a hospital diagnosis code for rhabdomyolysis identifies patients with a median peak creatine kinase level of 1835 (interquartile range [IQR] 680 to 3986) IU/L, whereas the absence of such a code indicates patients without a creatine kinase level or with a measured median level of 130 (IQR 60 to 368) IU/L (unpublished data).

† Validation of Ontario Diabetes Database was performed on 1,225,218 patients and found to have a sensitivity of 86-90% and specificity of 92-97% for the diagnosis of diabetes mellitus ^[1]^

‡ Validation of acute kidney injury was performed on 39,500 hospitalizations with linked laboratory values. A code for acute kidney injury identifies a median absolute increase in serum creatinine of 98 μmol/L (IQR, 43 to 200 μmol/L) from the most recent value before hospitalization, and the absence of such code represents a median increase of 6 μmol/L (IQR, -4 to 20 μmol/L) ^[2]^

§ Has a sensitivity of 94% and a positive predictive value of 100% ^[3]^

**Table D. Reasons the observation time was censored amongst the matched cohort.**

|  | **Number of Censoring Events**  (rate per 10,000 person-years) | | **Standardized Difference** | **P-value** |
| --- | --- | --- | --- | --- |
|  | **Chinese**  n = 19,033 | **Non-Chinese**  n = 57,099 |  |  |
| End of follow-up (March 31, 2013) | 3,009 (1239.8) | 6,954 (1127.2) | 10% | 0.6933 |
| Death * | 324 (133.5) | 965 (156.4) | 0% | 0.0071 |
| Statin discontinuation † | 15,700 (6469.0) | 49,180 (7971.5) | 10% | <0.0001 |

* Assessed using vital status field of Registered Persons Database of Ontario, which has a sensitivity of 94% and a positive predictive value of 100% ^[3]^

† Statin discontinuation was defined by no evidence of a repeat prescription for the same statin within 30 days following the end of the previous prescription day supply.

**Table E. Statin-associated adverse outcomes in Chinese and non-Chinese (referent) adjusting for competing risk of death.**

|  | **Number of Events**  (rate per 10,000 person-years) | | **Hazard Ratio**  (95% CI) | **P-value** |
| --- | --- | --- | --- | --- |
|  | **Chinese**  n= 19,033 | **Non-Chinese**  n=57,099 |  |  |
| **Rhabdomyolysis *** | 16 (6.6) | 79 (12.8) | 0.55 (0.30 to 1.02) | 0.06 |
| **Incident diabetes** † | 182 (267.5) | 475 (252.4) | 1.02 (0.85 to 1.23) | 0.82 |
| **Acute kidney injury *** | 215 (89.2) | 556 (90.8) | 0.93 (0.30 to 1.02) | 0.06 |

***Abbreviations***: confidence interval (CI)

* Outcomes assessed with hospital diagnosis codes. This underestimates the true event rate because these codes have high specificity but low sensitivity.

† Diabetes diagnoses assessed with the Ontario Diabetes Database; only matched sets where none of the users had evidence of baseline diabetes were included in this analysis (6,170 Chinese, 18,510 non-Chinese).

|  | **Number (proportion) with at least one outpatient blood test in follow-up** | | **Standardized Difference** |  |
| --- | --- | --- | --- | --- |
|  | **Chinese**  n= 19,033 | **Non-Chinese**  n=57,099 |  |  |
| Creatine kinase | 7530 (39.6%) | 21634 (37.9%) | 3% |  |
| Serum creatinine | 9430 (49.6%) | 27118 (47.5%) | 4% |  |
| Serum glucose | 9236 (48.5%) | 27147 (47.5%) | 2% |  |
| Glycated hemoglobin | 6578 (34.6%) | 17685 (31.0%) | 8% |  |

**Table F: Outpatient blood testing during follow-up.**

**Table G. Statin-associated adverse outcomes in Chinese and non-Chinese (referent) with at least 100 days grace period for subsequent statin prescription.**

|  | **Number of Events**  (rate per 10,000 person-years) | | **Hazard Ratio**  (95% CI) | **P-value** |
| --- | --- | --- | --- | --- |
|  | **Chinese**  n= 19,033 | **Non-Chinese**  n=57,099 |  |  |
|  |  |  |  |  |
| **Rhabdomyolysis *** | 30 (6.1) | 190 (13.5) | 0.55 (0.35 to 0.87) | 0.01 |
| **Incident diabetes** † | 368 (261.9) | 1010 (234.8) | 1.14 (0.99 to 1.32) | 0.07 |
| **Acute kidney injury *** | 381 (78.1) | 1281(92.0) | 0.86 (0.75 to 1.00) | 0.04 |

***Abbreviations***: confidence interval (CI)

* Outcomes assessed with hospital diagnosis codes. This underestimates the true event rate because these codes have high specificity but low sensitivity.

† Diabetes diagnoses assessed with the Ontario Diabetes Database; only matched sets where none of the users had evidence of baseline diabetes were included in this analysis (6,170 Chinese, 18,510 non-Chinese).

**Figure A. Flow diagram of cohort selection.**

Chinese and non-Chinese new statin users between June 2002 and March 2013

**(n = 1,343,058)**

**Patients excluded from study (n = 717,285)**

Age <66 at the time of any prescription: 71,484

Statin use in 180 days prior to index date: 430,021

No evidence of eligibility of healthcare in 3 years prior to prescription date: 152,609

Use of strong CYP3A4 inhibitors on or in 180 days prior to index date: 63,171

**Patients eligible for matching**

**(n = 625,773)**

Chinese users: 20,598

Non-Chinese users: 605,175

**Patients after matching**

**(n = 76,132)**

Chinese users: 19,033

Non-Chinese users: 57,099

**Figure B. Total number of Chinese newly dispensed rosuvastatin each year and proportion with ≥10mg/day starting dose.**


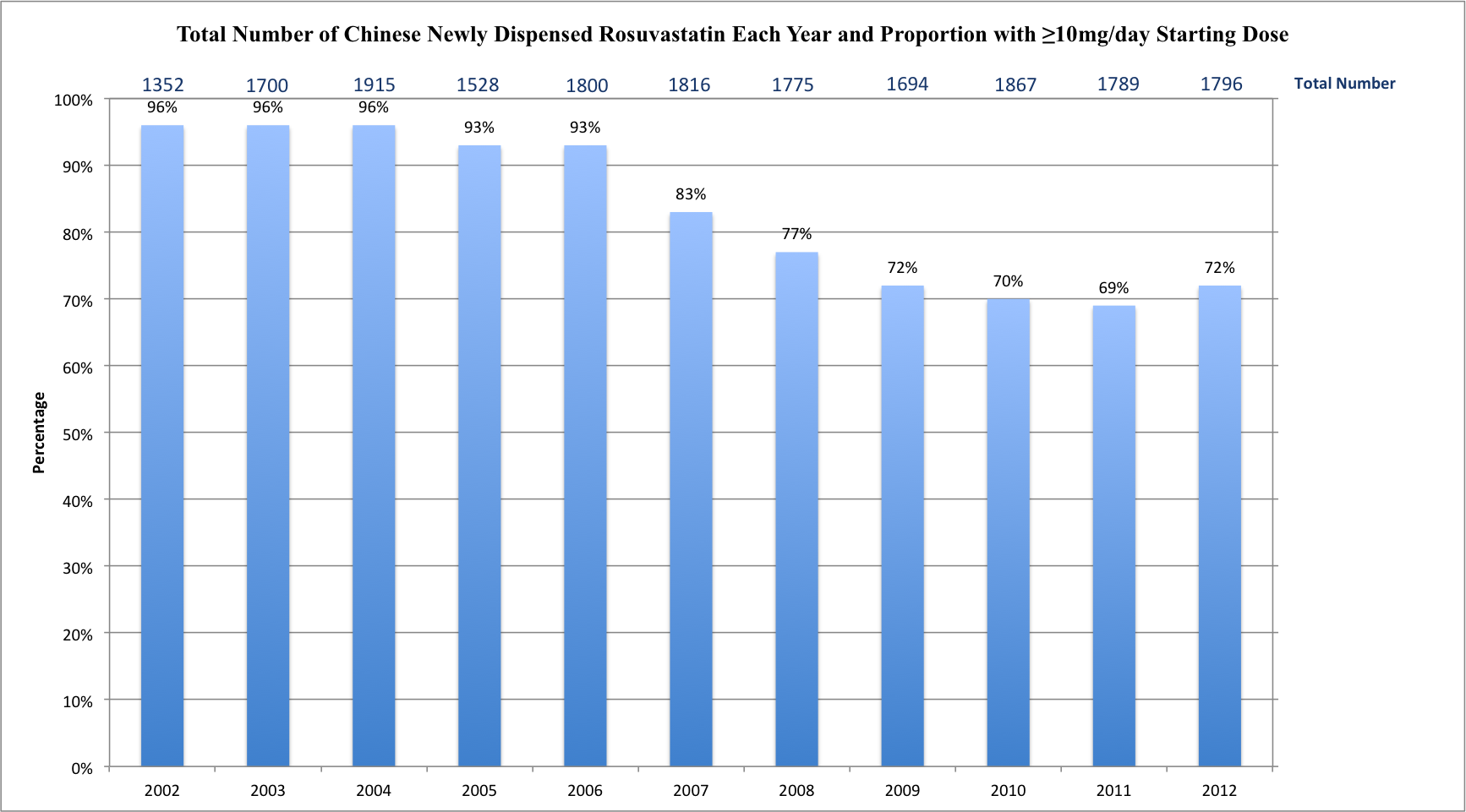


**REFERENCES**

1. Hux JE, Ivis F, Flintoft V, Bica A. Diabetes in Ontario: determination of prevalence and incidence using a validated administrative data algorithm. *Diabetes Care.* 2002; 25:512-6.

2. Hwang Y, Shariff S, Gandhi S, Wald R, Clark E, Fleet J, Garg AX. Validity of the International Classification of Diseases, Tenth Revision code for acute kidney injury in elderly patients at presentation to the emergency department and at hospital admission. *BMJ Open*. 2012;2(6).

3. Jha P, Deboer D, Sykora K, Naylor C. Characteristics and mortality outcomes of thrombolysis trial participants and nonparticipants: a population-based comparison. *Journal of the American College of Cardiology*. 1996; 27 (6): 1335-1342.
